# The value of restriction spectrum imaging in predicting lymph node metastases in rectal cancer: a comparative study with diffusion-weighted imaging and diffusion kurtosis imaging

**DOI:** 10.1186/s13244-024-01852-z

**Published:** 2024-12-19

**Authors:** Huijia Yin, Wenling Liu, Qin Xue, Chen Song, Jipeng Ren, Ziqiang Li, Dongdong Wang, Kaiyu Wang, Dongming Han, Ruifang Yan

**Affiliations:** 1 Department of MR, The First Affiliated Hospital, Xinxiang Medical University, Weihui, China; 2 Hematology Laboratory, The First Affiliated Hospital of Xinxiang Medical University, Xinxiang, China; 3 Department of Radiology, People’s Hospital of Zhengzhou, Zhengzhou, 450000 PR China; 4 MR Research China, GE Healthcare, Beijing, China

**Keywords:** Rectal cancer, Lymph node metastases, Restrictive spectrum imaging, Diffusion kurtosis imaging, Diffusion-weighted imaging

## Abstract

**Background:**

To investigate the efficacy of three-compartment restriction spectrum imaging (RSI), diffusion kurtosis imaging (DKI), and diffusion-weighted imaging (DWI) in the assessment of lymph node metastases (LNM) in rectal cancer.

**Methods:**

A total of 77 patients with rectal cancer who underwent pelvic MRI were enrolled. RSI-derived parameters (f_1_, f_2_, and f_3_), DKI-derived parameters (D_app_ and K_app_), and the DWI-derived parameter (ADC) were calculated and compared using a Mann–Whitney *U* test or independent samples *t*-test. Logistic regression (LR) analysis was used to identify independent predictors of LNM status. Area under the receiver operating characteristic curve (AUC) and Delong analysis were performed to assess the diagnostic performance of each parameter.

**Results:**

The LNM-positive group exhibited significantly higher f_1_ and K_app_ levels and significantly lower f_3_, D_app_, and ADC levels compared to the LNM-negative group (*p* < 0.05). There was no difference in f_2_ levels between the two groups (*p* = 0.783). LR analysis showed that D_app_ and K_app_ were independent predictors of a positive LNM status. AUC and Delong analysis showed that DKI (D_app_ + K_app_) exhibited significantly higher diagnostic efficacy (AUC = 0.908; sensitivity = 87.10%; specificity = 86.96%) than RSI (f_1_ + f_3_) and DWI (ADC), with AUCs were 0.842 and 0.771 (*Z* = 2.113, 3.453; *p* = 0.035, < 0.001, respectively). The AUC performance between RSI and DWI was also statistically significant (*Z* = 1.972, *p* = 0.049).

**Conclusion:**

The RSI model is superior to conventional DWI but inferior to DKI in differentiation between LNM-positive and LNM-negative rectal cancers. Further study is needed before it could serve as a promising biomarker for guiding effective treatment strategies.

**Critical relevance statement:**

The three-compartment restriction spectrum imaging was able to differentiate between LNM-positive and LNM-negative rectal cancers with high accuracy, which has the potential to serve as a promising biomarker that could guide treatment strategies.

**Key Points:**

- Three-compartment restriction spectrum imaging could differentiate lymph node metastases in rectal cancer.
- Diffusion kurtosis imaging and diffusion-weighted were associated with lymph node metastases in rectal cancer.
- The combination of different parameters has the potential to serve as a promising biomarker.

**Graphical Abstract:**

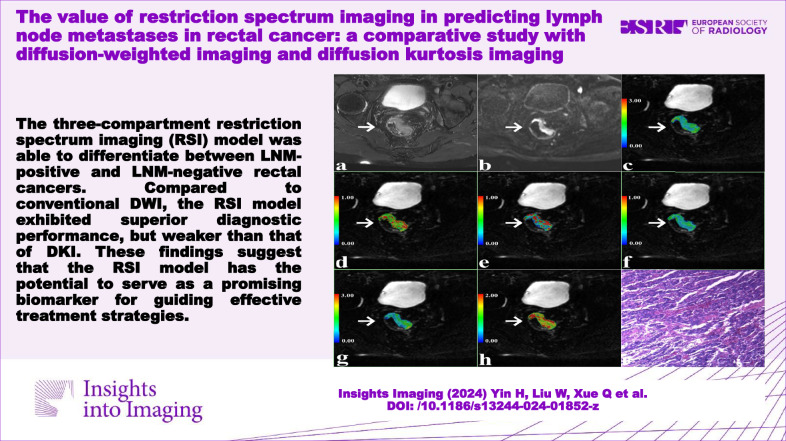

## Introduction

Rectal cancer has emerged as the fourth most prevalent form of cancer worldwide and the second leading cause of mortality [1, 2]. Clinical interventions for rectal cancer, such as surgical excision and neoadjuvant chemoradiotherapy, can lead to significant adverse events in the absence of a precise assessment of lymph node metastasis (LNM) status [3, 4]. Smith et al demonstrated that LNM was correlated with a poorer prognosis, even in cases of complete regression of the primary tumor, and the rate of recurrence was notably higher in LNM-positive patients compared to LNM-negative [5]. Therefore, accurate preoperative evaluation of LNM is essential for guiding treatment strategies and improving clinical outcomes in individuals with rectal cancer.

Currently, clinical practitioners rely on highly invasive biopsy procedures to determine the LNM status of rectal cancer, which carries risks and complications. Magnetic resonance imaging (MRI) has been widely recommended as a non-invasive tool to evaluate LNM status [6]. However, morphological assessment has been associated with low sensitivity and specificity [7]. Diffusion-weighted imaging (DWI) is a well-known diffusion MRI technique, and several studies have shown that its quantitative parameter, the apparent diffusion coefficient (ADC), has a positive role in the differential diagnosis of LNM in rectal cancer [8, 9]. However, DWI relies on accurately measuring the diffusion motion of water molecules using Gaussian distribution, limiting the diagnostic accuracy [10, 11]. Diffusion kurtosis imaging (DKI), another diffusion MRI technique based on the theory of a non-Gaussian distribution of water molecules in tissues, was first proposed by Jenson et al in 2005 [12]. In contrast to traditional DWI, DKI comprehensively accounts for the complex nature of water molecule diffusion within tissues by incorporating fourth-order 3-dimensional tensors into the original diffusion imaging model. This improves the precision in the quantitative evaluation of diffusion characteristics in tissues, thereby capturing the nuanced complexity of tissue microstructure with increased sensitivity [13, 14]. Currently, few studies have directly compared the differences in DKI-related parameters between metastatic and non-metastatic lymph nodes in rectal cancer from the perspective of the primary lesion, presenting challenges in the development of a comprehensive reference for clinical diagnosis and treatment [15, 16].

Restriction spectrum imaging (RSI) is a cutting-edge diffusion model employed in MRI that effectively categorizes water diffusion into distinct microscopic tissue compartments, such as restricted, hindered, and free water compartments, by fitting signals to a linear combination of diffusion-weighted models [17]. To date, RSI has demonstrated initial promise in the evaluation of various diseases, including prostate cancer [18] and breast cancer [19]. But to our knowledge, in the field of rectal cancer, only Xiong et al have assessed tumor grading using RSI [20].

Therefore, this study aims to explore the diagnostic value of three-compartment RSI in the assessment of LNM in rectal cancer, and compare it with DKI and DWI, with a view to providing novel imaging markers for accurate clinical diagnosis and to guide treatment strategies to improve clinical outcomes.

## Materials and methods

### Study population

**Fig. 1:**
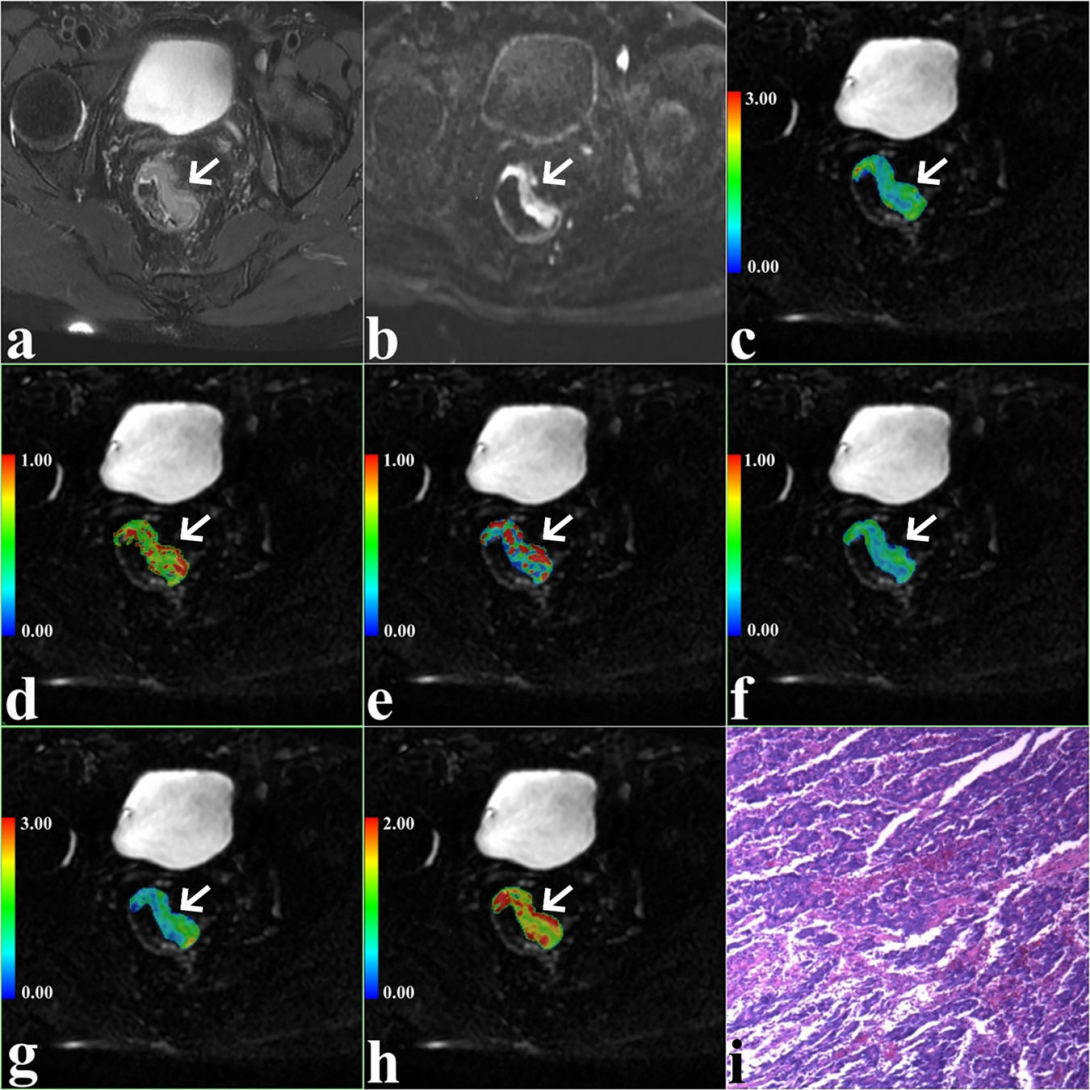
A 67-year-old man with LNM-positive rectal cancer in the left wall of rectum (arrow, pseudo colored region). T2-weighted image showed a slightly hyperintensity mass (**a**) with restricted diffusion on DWI (**b**) ADC map (**c**). **d**–**h** f_1_, f_2_, f_3_, D_app_, and K_app_ maps of the same slice as in **a**–**c**. **i** Pathological image (original magnification, ×100)

The current study was approved by the local ethics committee, and all participants provided written informed consent. Between March 2023 and May 2024, a total of 100 patients underwent pelvic MRI due to suspected rectal cancer following clinical evaluation. The exclusion criteria were as follows: (1) patients with pathologically confirmed non-rectal cancer (*n* = 5); (2) patients with inconclusive pathological results (*n* = 4); (3) patients with a time interval of more than 2 weeks between scanning and biopsy (*n* = 3); (4) patients who did not complete all MRI scans or whose images were of insufficient quality for analysis (*n* = 6); and (5) patients who had undergone relevant treatment prior to the scans (*n* = 5). Consequently, 77 patients were enrolled in the study (Fig. 1). Patient characteristics, including age, gender, maximum tumor diameter, and CEA levels, were recorded.

### Image acquisition

A 3.0-Tesla MRI system (Signa Architect, GE Medical Systems, Milwaukee, WI) equipped with a 16-channel phased-array body coil was utilized for pelvic imaging. Prior to the scan, all patients had their rectums emptied and were given anti-peristalsis medication when appropriate. All patients were placed in the supine position, feet-first into the scanner. Initially, a T2-weighted imaging (T2WI) sequence in the axial plane was conducted to delineate the tumor location that employed the following parameters: repetition time/echo time (TR/TE) = 4600/125 ms; slice thickness = 3 mm; gap = 0.3 mm; number of excitations (NEX) = 2; field of view (FOV) = 360 × 360 mm; and matrix = 268 × 320. The T2WI resulted in a total scan time of 2 min 49 s. Subsequently, using T2WI as a reference, multiple b-value sequences were performed for the slices containing the lesions. The scanning position, layer thickness, and gap of the b-value sequences remained unchanged, and the following parameters were used: TR/TE = 445/85.3 ms; slice thickness = 3 mm; gap = 0.3 mm; b-values = 0, 50, 100, 150, 200, 400, 600, 800, 1000, 1500, and 2000 s/mm^2^; NEX = 1, 1, 1, 1, 1, 2, 2, 2, 4, 4, and 4; FOV = 360 × 360 mm; and matrix 128 × 128. This resulted in a total scan time of 4 min 55 s.

### Parameter generation

All analyses were performed using Matlab R2018b (MathWorks Inc., Natick, MA, USA). Preceding the quantitative analysis, the raw data from the b-value sequences underwent various corrections to address B_0_ distortion, gradient nonlinearities, and eddy current distortions. The DWI is expressed using the following equation:

1 $${{{{\rm{S}}}}}_{{{{\rm{b}}}}}/{{{{\rm{S}}}}}_{0}=\exp \left(-{{{\rm{b}}}}\times {{{\rm{ADC}}}}\right)$$

where ADC is the apparent diffusion coefficient, b is the diffusion sensitizing factor, and S_0_ and S_b_ are the signal intensities under different b-values (0 s/mm^2^ and 800 mm^2^/s, respectively) [8].

The DKI is expressed using the following equation:

2 $${{{{\rm{S}}}}}_{{{{\rm{b}}}}}={{{{\rm{S}}}}}_{0}\times \exp \left(-{{{\rm{b}}}}\times {{{{\rm{D}}}}}_{{{{\rm{app}}}}}+{{{{\rm{b}}}}}^{2}\times {{{{\rm{D}}}}}_{{{{\rm{app}}}}}^{2}\times {{{{\rm{K}}}}}_{{{{\rm{app}}}}}/6\right)$$

where K_app_ is kurtosis, representing the deviation from the Gaussian distribution, while D_app_ is diffusivity, representing the diffusion coefficient corrected for non-Gaussian bias [12].

The RSI is expressed using the following equation:

3 $$S(b)={f}_{1}{e}^{-bD1}+{f}_{2}{e}^{-bD2}+{f}_{3}{e}^{-bD3},D1 \, < \, D2 \, < \, D3$$

where f_1_, f_2_, and f_3_ are the volume fractions of restricted diffusion, hindered diffusion, and free water diffusion compartments, respectively, and D1, D2, and D3 are the ADCs of the corresponding compartments. To prevent overfitting, ensure the linearization of the RSI model, and maintain comparability of volume fractions across compartments, according to the theoretical values and experimental findings, D1, D2, and D3 were standardized to 0.5 × 10^−3^ mm^2^/s, 1.3 × 10^−3^ mm^2^/s, and 3.0 × 10^−3^ mm^2^/s, respectively [20].

All the parameters included in this study were derived from the primary tumor. Two independent radiologists who were blinded to histopathologic and clinic data determined the whole-tumor volume by manually analyzing regions of interest (ROIs) along the tumor’s outer edge on DWI images using ITKSNAP software (version 3.8.0; http://www.itksnap.org). Obvious cystic, necrotic, hemorrhagic, and calcified regions were avoided by referencing the corresponding T2WI images. Subsequently, the whole-tumor ROIs were automatically transferred to the parametric maps (including ADC, D_app_, K_app_, f_1_, f_2_, and f_3_), followed by the calculation of parametric values.

### Histopathological evaluation

All specimens were obtained by surgical resection of the primary tumor and nodal dissection, and the median interval from MRI examination to surgery was 11 days (1–14 days). All resected samples were fixed in formalin, dehydrated, immersed in wax, embedded in paraffin, sectioned, and stained with hematoxylin and eosin (H&E). Pathologic staging was performed according to the guidelines outlined in the Eighth Edition American Joint Committee on Cancer Staging Manual [21]. Patients with one or more lymph node metastases were assigned to the LNM-positive group, otherwise the LNM-negative group.

### Statistical analysis

Interclass correlation coefficients (ICC) were calculated to evaluate interobserver agreement for DKI, RSI, and DWI parameters, with ICCs > 0.75 indicating excellent reliability [22]. Differences between the LNM-positive and LNM-negative groups were analyzed using a Mann–Whitney *U* test, independent samples *t*-test, or chi-square test based on the distributional properties of the variables. The diagnostic performance of DKI, RSI, and DWI was evaluated using the area under the receiver operating characteristic curve (AUC). The deLong test was used to compare the differences in AUCs of each parameter. Logistic regression (LR) analysis was employed to identify independent influencing factors and combined diagnostic assessments. Statistical analyses were conducted using MedCalc software (version 15.0; MedCalc Software, Ostend, Belgium). *p*-values less than 0.05 were considered statistically significant.

## Results

### Patient characteristics

A total of 77 patients, 31 patients with LNM-positive and 46 LNM-negative rectal cancer were included. No statistically significant differences between the two groups were observed in age, gender, maximum diameter, or CEA levels (*p* > 0.05, Table 1).

**Table 1:** Comparison of different variables among different groups

Variables	LNM-positive (*n* = 31)	LNM-negative (*n* = 46)	t / χ^2^ / *z* value	*p*-value
Age (years)*	63.54 ± 10.03	60.68 ± 10.85	1.171	0.246^a^
Maximum diameter (cm)*	4.24 ± 1.39	3.83 ± 1.26	−1.298	0.199^a^
Sex, *n* (%)			2.542	0.111^c^
Male	16 (51.61%)	32 (69.57%)		
Female	15 (48.39%)	14 (30.43%)		
CEA (ng/mL)^#^	4.08 (1.84, 13.35)	2.68 (1.21, 6.02)	−1.828	0.068^b^
f_1_^#^	0.43 (0.35, 0.50)	0.21 (0.09, 0.38)	−5.027	< 0.001^b^
f_2_^#^	0.12 (0.07, 0.19)	0.13 (0.06, 0.22)	−0.275	0.783^b^
f_3_^#^	0.27 (0.20, 0.30)	0.36 (0.28, 0.55)	−3.454	0.001^a^
D_app_ (× 10^−3^mm^2^/s)*	1.04 ± 0.44	1.85 ± 0.07	5.802	< 0.001^a^
K_app_*	0.77 ± 0.27	0.49 ± 0.18	−5.322	< 0.001^a^
DWI / ADC (× 10^−3^mm^2^/s)*	1.09 ± 0.29	1.57 ± 0.62	4.550	< 0.001^a^

*LNM* lymph node metastasis

* Data are means ± SDs

^#^ Data are medians, with IQRs in parentheses

^a^ Independent *t*-test

^b^ Chi-squared test

^c^ Mann–Whitney *U* test

### Consistency evaluation

The measurements of D_app_, K_app_, ADC, f_1_, f_2_, and f_3_ by the two observers showed excellent consistency, with all ICC values exceeding 0.75. The average readings from both observers were utilized for subsequent analysis.

### Parameter comparison

**Fig. 2:**
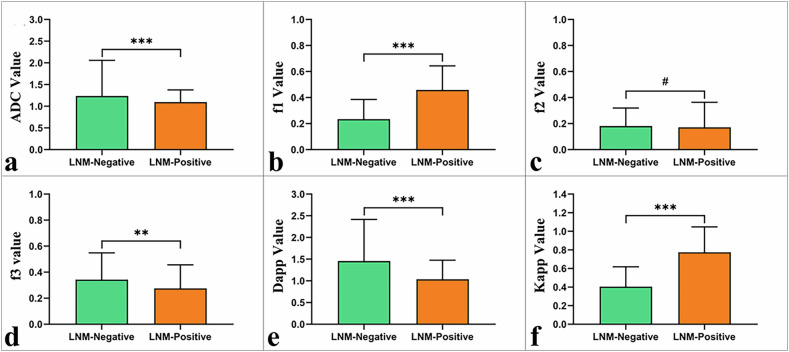
Boxplots of various parameters in LNM-positive and LNM-negative rectal cancer: **a** ADC; **b** f_1_; **c** f_2_; **d** f_3_; **e** D_app_; and **f** K_app_. ***p < 0.001, **p < 0.01, and #*p* > 0.05

The LNM-positive group exhibited significantly higher f_1_ and K_app_ values and significantly lower f_3_, D_app_, and ADC values compared to the LNM-negative group (*p* < 0.05). However, the disparity in f_2_ values between the two groups did not reach statistical significance (*p* = 0.783; Table 1; Fig. 2).

### LR analysis

Age, gender, maximum diameter, CEA levels, D_app_, K_app_, ADC, f_1_, f_2_, and f_3_ were all included in the LR analysis. Univariate analysis demonstrated that CEA levels, f_1_, f_3_, D_app_, K_app_, and ADC were all independent predictors of LNM status (aP < 0.05). Further multivariate analysis demonstrated that only D_app_ (*p* = 0.034) and K_app_ (*p* = 0.004) were significantly correlated with a positive LNM status in rectal cancer patients (Table 2).

**Table 2:** Univariate and multivariate analyses

Parameters	Univariate analyses		Multivariate analyses	
	OR* (95% CI)	*p*-value	OR* (95% CI)	*p*-value
Age (years)	0.755 (0.473–1.205)	0.239	/	/
Sex	0.467 (0.182–1.199)	0.113	/	/
Maximum diameter (mm)	1.365 (0.857–2.173)	0.190	/	/
CEA (ng/mL)	3.084 (0.868–10.959)	0.082	2.586 (0.283–23.657)	0.400
f_1_	6.974 (2.703–17.994)	< 0.001	0.773 (0.126–4.754)	0.781
f_2_	1.062 (0.675–1.673)	0.794	/	/
f_3_	0.411 (0.220–0.767)	0.005	0.459 (0.174–1.207)	0.114
D_app_ (× 10^−3^mm^2^/s)	0.104 (0.034–0.318)	< 0.001	0.065 (0.005–0.814)	0.034
K_app_	17.780 (4.646–68.043)	< 0.001	12.989 (2.224–75.854)	0.004
ADC (× 10^−3^mm^2^/s)	0.194 (0.074–0.513)	0.001	5.220 (0.987–27.607)	0.052

All factors with *p* < 0.1 in univariate analyses were included in multivariate regression analyses

*OR* odds ratio, *CI* confidence interval

* OR for per 1 standard deviation

### Diagnostic performance

**Fig. 3:**
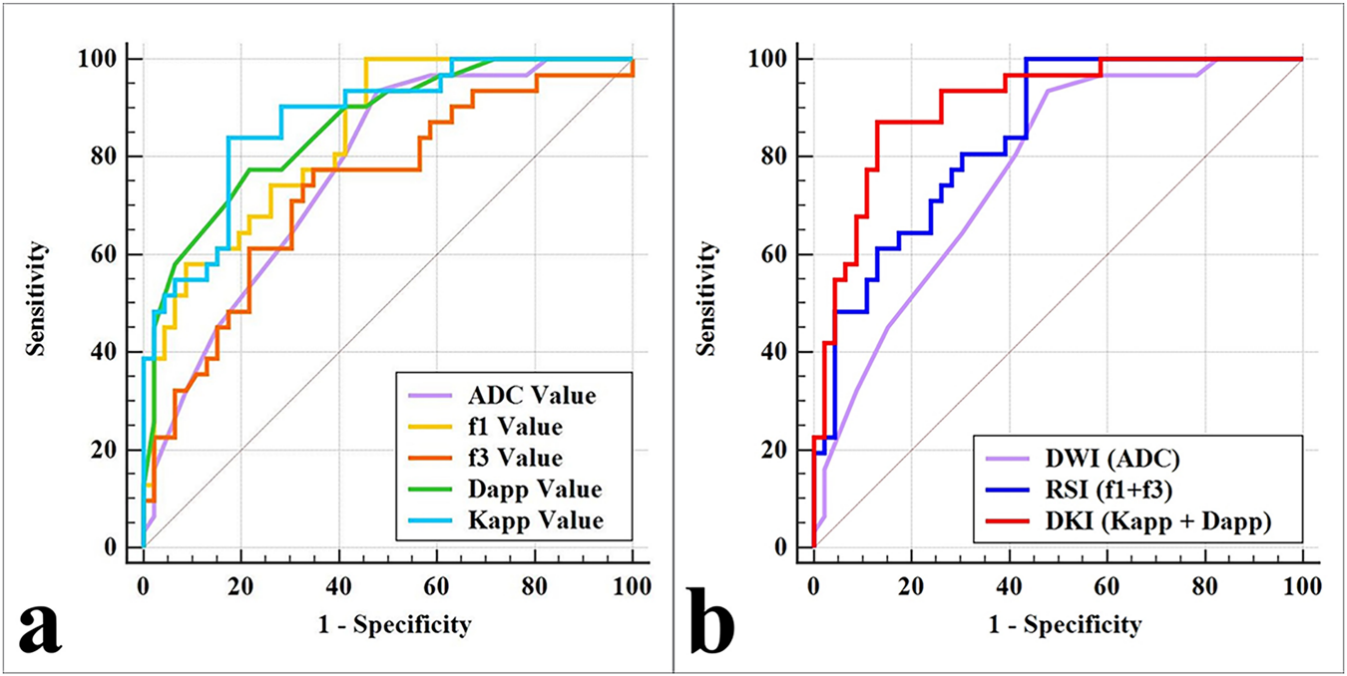
The areas under receiver-operator characteristic (ROC) curves of different parameters (**a**) and different diffusion models (**b**)

Among the included parameters, K_app_ demonstrated the highest diagnostic efficacy (AUC = 0.874; sensitivity = 83.87%; specificity = 82.61%). The AUCs for D_app_, f_1_, ADC, and f_3_ were 0.860, 0.839, 0.771, and 0.733, respectively. There is a statistically significant difference in AUCs between K_app_ and ADC (*Z* = 2.047, *p* = 0.041), and between K_app_ and f_3_ (*Z* = 2.131, *p* = 0.033). Among the different diffusion models, DKI (D_app_ + K_app_) exhibited the highest diagnostic efficacy (AUC = 0.908; sensitivity = 87.10%; specificity = 86.96%), which was significantly higher than RSI (f_1_ + f_3_) and DWI (ADC) (AUC = 0.842, 0.771; *Z* = 2.113, 3.453; and *p* = 0.035, < 0.001, respectively). Furthermore, there was a significant difference in AUC between RSI and DWI (*Z* = 1.972, *p* = 0.049). The diagnostic performances are summarized in Fig. 3 and Table 3.

**Table 3:** Predictive performance of different variables

Variables	AUC (95% CI)	*p*-value	Cutoff	Sensitivity	Specificity	Comparison with DKI
f_1_	0.839 (0.738–0.913)	< 0.001	0.233	96.77%	54.35%	*Z* = 2.360, *p* = 0.018
f_2_	0.519 (0.402–0.634)	0.784	/	/	/	/
f_3_	0.733 (0.620–0.828)	< 0.001	0.305	77.42%	65.22%	*Z* = 2.825, *p* = 0.005
D_app_ (× 10^−3^mm^2^/s)	0.860 (0.762–0.929)	< 0.001	1.200	77.42%	78.26%	*Z* = 1.657, *p* = 0.098
K_app_	0.874 (0.779–0.939)	< 0.001	0.616	83.87%	82.61%	*Z* = 1.499, *p* = 0.134
DWI/ADC (× 10^−3^mm^2^/s)	0.771 (0.661–0.859)	< 0.001	1.400	93.55%	52.17%	*Z* = 3.453, *p* < 0.001
RSI	0.842 (0.741–0.915)	< 0.001	/	96.77%	56.52%	*Z* = 2.113, *p* = 0.035
DKI	0.908 (0.820–0.962)	< 0.001	/	87.10%	86.96%	/

DWI = ADC, RSI = f_1_ + f_3_, DKI = D_app_ + K_app_

*CI* confidence interval

## Discussion

In this study, we demonstrated that the RSI-derived parameters (f_1_, f_3_), DKI-derived parameters (D_app_, K_app_), and DWI-derived parameter (ADC) could be used to distinguish LNM-positive from LNM-negative rectal cancer. The multivariate analysis showed that D_app_ and K_app_ were independent factors correlated with a positive TMN status. In addition, the DKI (D_app_ + K_app_) model exhibited optimal diagnostic efficacy, which was significantly higher than those of the RSI (f_1_ + f_3_) and DWI (ADC) models, and the diagnostic efficacy of RSI (f_1_ + f_3_) model was significantly higher than DWI model.

DWI is a classical MRI diffusion imaging technique that models the diffusion of water molecules in biological tissues as a uniform Gaussian distribution [23]. In this study, the ADC values of LNM-positive patients were significantly lower than those of LNM-negative patients. These results are in line with previous studies that found that reduced ADC values were correlated with restricted internal water molecules leading to higher malignancy and more tightly organized structures in LNM-positive lesions [8, 9]. Therefore, the results of the present study provide evidence of the value of DWI in diagnosing the LNM status of rectal cancer.

Three-compartment RSI classifies the diffusion of water molecules in biological tissues into diffusion (f_1_), hindered diffusion (f_2_), and free-water diffusion (f_3_) [18]. The f_1_ refers to the retention of water molecules within a restricted space, f_2_ denotes the passage delay of smaller molecules when they traverse a cellular impediment, and f_3_ delineates the random motion of water molecules in the absence of any obstacle [24]. The findings of the current study demonstrated the potential utility of RSI-derived parameters in differentiating between LNM-positive and LNM-negative rectal cancer. Specifically, LNM-positive rectal cancer exhibited a higher f_1_ and a lower f_3_ value compared to LNM-negative. This may be attributed to an increase in cellularity as the tumor becomes more malignant, leading to a rise in the volume fraction of restricted diffusion within the microenvironment and subsequently higher f_1_ values in LNM-positive rectal cancer patients [8, 20, 25]. Additionally, it has been reported that highly malignant LNM-positive tumors often display substantial necrosis and reduced extracellular space, which could hinder water proton diffusion and lead to lower f_3_ values [19]. Furthermore, this study found no direct relationship between f_2_ and LNM status in rectal cancer. This finding may be attributed to f_2_’s lack of specificity in describing the cellularity of the tumor [20].

DKI quantitatively measures the complexity of tissue microstructure (K_app_) and the diffusion of water molecules within the tissues (D_app_) [26, 27]. DKI has demonstrated clinical utility in differentiating LNM-positive from LNM-negative in other cancers, such as breast cancer [28] and cervical cancer [29]. Similar to evaluation in different cancers, the analysis of the current study revealed that LNM-negative rectal cancer patients exhibited a decrease in K_app_ and an increase in D_app_ compared to the LNM-positive group. Variations in tissue malignancy levels may significantly contribute to these results. Compared to LNM-negative tumors, LNM-positive tumors are more compact and exhibit characteristics such as hemorrhaging, necrosis, and higher tissue heterogeneity [28, 29]. These malignant characteristics impede the rate of water molecule diffusion and increase the deviation of the diffusion movement of water molecules, resulting in a decline in the D_app_ value and an increase in the K_app_ value.

This study compared the diagnostic performance of DKI, RSI, and DWI in assessing the LNM status of rectal cancer patients. The results showed that DKI had higher diagnostic performance than RSI, followed by DWI. The DWI model exhibited the lowest diagnostic efficacy as it assumes a uniform Gaussian distribution of water molecules which does not effectively capture the complex and heterogenous nature of biological tissues due to variation in factors such as cellularity and tissue structure. The RSI model, in contrast to DWI, attempts to describe water diffusion as restricted, hindered and free diffusion, and thereby provides a more comprehensive picture of water molecule diffusion [19, 20]. As previously discussed, DKI is more sensitive to the diffusion motion of water molecules in tissues with a non-Gaussian distribution and measures cellularity and tissue structure changes. This enables an accurate and more realistic representation of the complex characteristics of rectal cancer lesions [26, 27].

Selecting the b-value is one of the most important factors that influences the diagnostic performance of the RSI model. Some reports have indicated that RSI fitted using 4–7 b-values with a maximum of 4000 mm^2^/s maintained superior performance [30, 31]. However, Felker et al demonstrated that a combination of fewer and smaller b-values (four b-values up to a maximum of 1400 mm^2^/s) could also be employed and a reliable RSI fit could be achieved [32]. This study selected twelve b-values up to 2000 mm^2^/s to optimize scanning time and image quality to fit the RSI. Similar to a study by Xiong et al [20], this study showed fair diagnostic performance of RSI, providing evidence that the selected b-value was a reliable tool in assessing rectal cancer. However, there is currently no consensus on the optimal b-value for RSI model fitting, and further studies with larger sample sizes are needed to establish standardized protocols.

The present study has some limitations. First, it was conducted at a single institution with a limited number of participants. Second, the RSI, DKI, and DWI were all based on echo planar imaging, resulting in a lack of visualization of small lesions. Finally, the choice of b-value for RSI images may require further optimization to ensure optimal image quality and diagnostic accuracy.

## Conclusion

The three-compartment RSI model was able to differentiate between LNM-positive and LNM-negative rectal cancers. Compared to conventional DWI, the RSI model exhibited superior diagnostic performance, but weaker than that of DKI. These findings suggest that the RSI model has the potential to serve as a promising biomarker for guiding effective treatment strategies.

## Data Availability

All data are with the corresponding author and can be obtained by mail if necessary.

## Abbreviations

ADC: Apparent diffusion coefficient
AUC: Area under the receiver operating characteristic curve
DKI: Diffusion kurtosis imaging
DWI: Diffusion-weighted imaging
LNM: Lymph node metastases
LR: Logistic regression
MRI: Magnetic resonance imaging
RSI: Restriction spectrum imaging
